# Impulsive and compulsive behaviors can be induced by opposite GABAergic dysfunctions inside the primate ventral pallidum

**DOI:** 10.3389/fnsys.2022.1009626

**Published:** 2022-12-08

**Authors:** Yosuke Saga, Laurent Galineau, Léon Tremblay

**Affiliations:** ^1^Institut des Sciences Cognitives Marc Jeannerod, UMR-5229 CNRS, Bron Cedex, France; ^2^UMR INSERM U1253, Université François Rabelais de Tours, Tours, France; ^3^Université Claude-Bernard Lyon1, Villeurbanne, France

**Keywords:** non-human primate, anterior pallidum, muscimol, bicuculline, anxiety behavior, hyperactivity state

## Abstract

**Introduction:** The ventral pallidum (VP) is central in the limbic Basal Ganglia circuit, controlling both appetitive (approach) and aversive (avoidance) motivated behaviors. Nevertheless, VP involvement in pathological aspects remains unclear, especially in the behavioral expression of different motivational dysfunctions. This study aimed to investigate how the VP contributes to the expression of abnormal behaviors *via* opposite GABAergic dysfunctions.

**Methods:** Opposite GABAergic dysfunctions were induced by injecting muscimol (a GABA_A_ agonist) and bicuculline (a GABA_A_ antagonist) into monkeys. We determined the effects of both substances on self-initiated behaviors in lab-chair and in free-moving home-cage contexts in six monkeys, and in two animals performing an approach-avoidance task in appetitive and aversive contexts.

**Results:** While the self-initiated behaviors induced by bicuculline injections in VP were characterized by compulsive behaviors such as repetitive grooming and self-biting, muscimol injections induced impulsive behaviors including limb movements in a lab-chair context and exploration behaviors in a free-moving context. More specific behavioral effects were observed in the approach-avoidance task. The muscimol injections induced premature responses and erroneous screen touches, which characterize impulsive and attention disorders, while the bicuculline injections into the VP increased passive avoidance (non-initiated action) and task-escape in an aversive context, suggesting an anxiety disorder.

**Conclusions:** These results show that activating or blocking GABAergic transmission in the VP impairs motivated behaviors. Furthermore, the behavioral expressions produced by these opposite disturbances show that the VP could be involved in anxiety-driven compulsive disorders, such as OCD, as well as in impulsive disorders motivated by attention deficits or reward-seeking, as seen in ADHD or impulse control disorders.

## Introduction

The ventral pallidum (VP) is a small structure under the anterior commissure (AC), and, in primates, corresponds to the limbic part of the external Globus Pallidus (François et al., 2004; Haber and Knutson, 2010; Saga et al., 2017). The VP receives large projections from the ventral striatum known to be involved in different motivational disorders (Tremblay et al., 2015; Sgambato-Faure et al., 2016) and projects to the limbic territories of both Basal Ganglia (BG) outputs, the internal Globus Pallidus (GPi) and the Substantia nigrapars reticulata (SNr), as well as to the subthalamic nucleus (François et al., 2004; Karachi et al., 2005). Several studies in rodents have studied the effects of pharmacological modulations of VP neuronal activity and its control by the Nucleus Accumbens (NAc), the primate ventral Striatum (Chrobak and Napier, 1993; Johnson and Napier, 1997; Root et al., 2015; Kupchik and Prasad, 2021). It has been shown in rodents at the behavioral level that this GABAergic projection from the NAc to VP exerts control both on locomotor activity (Austin and Kalivas, 1989) and on processes related to reward seeking and aversion (Creed et al., 2016; Heinsbroek et al., 2020; Moaddab et al., 2021; Liu et al., 2022). Therefore, we hypothesized that the primate VP occupies a key position in the limbic cortico-BG circuit, controlling the flow of motivational information to select context-dependent appetitive and aversive motivated behaviors (Saga et al., 2016, 2019).

Although the VP is thought to be a key structure in incentive motivation in human (Pessiglione et al., 2007), it is difficult to determine the VP’s specific involvement in human behavioral disorders using brain imaging due to its small size and the difficulty in separating its activity from that of neighboring structures, such as the GPi and nucleus basalis or extended amygdala. As the VP contains a high density of GABA_A_ receptors (Richards et al., 1987), inactivation of VP activity *via* injections of muscimol (a GABA_A_ agonist) in non-human primates (NHP), mimicking reversible lesions, has been shown to alter reward-related behaviors (Tachibana and Hikosaka, 2012). In contrast, increased VP neuronal activity induced by injecting bicuculline (a GABA_A_ antagonist) into NHPs led to increased sensitivity to aversive contexts (Saga et al., 2016), and produced repetitive behaviors such as finger licking or biting by activating a neuronal network involved in anxiety (Grabli et al., 2004; Galineau et al., 2017). Thus, these studies show that opposite changes in GABAergic transmission can produce different kinds of motivated behaviors. However, how these opposite GABAergic dysfunctions can alter spontaneous behaviors, i.e., in free-moving behavior independent of the task context, remains unknown.

Thus, our objective was to better understand how inhibiting (*via* muscimol) and activating (*via* bicuculline) VP activity would impact behavioral expression in different experimental contexts. Our hypothesis was that the GABA_A_ agonist and antagonist would induce opposite effects which could be expressed differently depending on the experimental context, the free-moving context allowing behavioral expression more similar to human behavioral disorders. Moreover, based on our previous studies concerning disruptions to the Pallidum inside these different functional territories in primates, we hypothesize that muscimol injection would produce an impulsive profile, as in ADHD, and bicuculline a compulsive profile driven by a state of anxiety as observed in OCD. This could explain that a lesion or dysfunction of the Pallidum may be involved in these two neuropsychiatric disorders which are both frequently observed in Tourette’s syndrome (Tremblay et al., 2015). To test these hypotheses, we injected muscimol and bicuculline directly into the VP of NHPs and evaluated their spontaneous behavior in their home cage or in the experimental chair, either without being involved in a behavioral task or while performing an approach-avoidance instrumental task in appetitive and aversive contexts.

Contrary to expected, muscimol and bicuculline injections into the VP of NHPs similarly affected performance in approach-avoidance instrumental tasks, with only the error type differentiating the two opposite pharmacological injections. These were more clearly distinguished in the free-moving context in the home cage. Inhibiting VP activity *via* muscimol produced hyperactive behaviors that could be related to impulsive disorders, while activating VP activity *via* bicuculline produced compulsive behaviors characteristic of anxiety disorders. Taken together, these results suggest that opposite GABAergic dysfunctions in the VP similarly affect motivated behaviors but have different effects in a free-moving context, suggesting potential involvement of VP dysfunctions in human impulsive and compulsive disorders.

## Materials and Methods

### Animals and surgical procedure for VP injections

**Figure 1:**
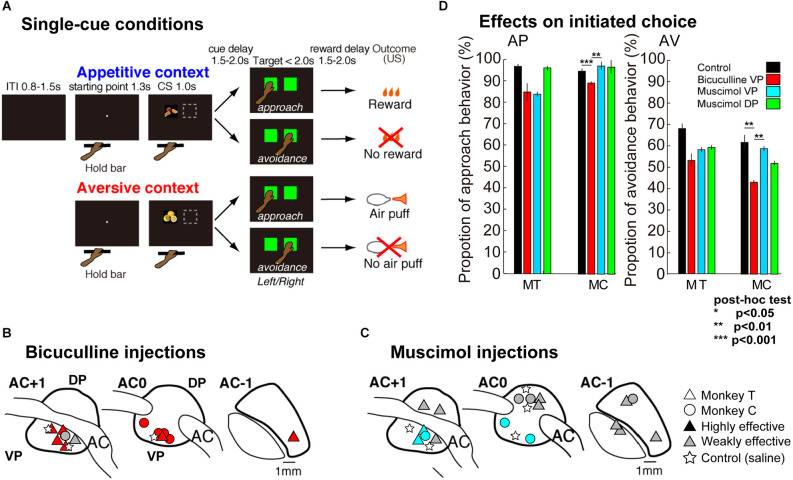
Description of the Appetitive-Aversive contexts of the delayed response task, and injection site locations. **(A)** A small white dot (starting point at visual angle of 0.4°) appeared at the center of the screen, when the monkey placed its left hand on the bar, after the inter-trial interval (0.8–1.5 s). The dot was replaced by a conditioned cue (CS, visual angle of 11°, single-cue condition). The CS was presented for 1.0 s either pseudo-randomly on the left or right side of the touch screen. The CSs provided the appetitive (AP) or aversive (AV) contexts; the monkeys could obtain a liquid reward (apple juice) or an aversive outcome (a puff of air) as unconditioned stimuli (USs) or avoid USs depending on their response. After presentation of the CSs, a random delay period of 1.5–2.0 s was introduced. Then, green square targets (visual angle of 12°) were presented for a maximum of 2.0 s on the left and right sides of the screen. The monkeys had to select one of the two targets by touching the screen. The targets disappeared as soon as one of them had been selected. If the monkeys selected the target at the position where the CS had been shown, either the liquid reward (appetitive CS approached) or the puff of air (aversive CS approached) occurred after a final delay of 1.5–2.0 s (reward delay). By contrast, if they selected the target at the other position, nothing happened; in other words, the monkeys missed out on the opportunity to earn a reward (appetitive CS avoided) or successfully prevented apuff of air (aversive CS avoided). **(B,C)** Location of bicuculline **(B)** and muscimol **(C)** injections while monkeys performed behavioral tasks. The black marks indicate the induction of more highly effective areas, with effects starting earlier, between 5 and 25 min. The gray marks indicate weaker effects, starting after 25–45 min. The circles and triangles indicate the injections in each monkey and the stars indicate a saline injection as a control. All injection sites are shown relative to the anterior commissure (AC). **(D)** Behavioral effects of injections on the rate of approach behavior in AP context (left) and avoidance behavior in AV context (right), the territory (VP or DP) and the injected agent (bicuculline or muscimol). Each color bar indicates the proportion of choice after injection (red: bicuculline into the VP, cyan: muscimol into the VP, green: muscimol into the DP) or control (black: saline) while performing tasks in the AP or AV contexts. The left and right panels show the behavioral performances of MT and MC, respectively. Asterisks inserted in the panel indicate the statistical significance. **p < 0.01, ***p < 0.001.

Eight monkeys were used in this study: six males *Macaca fascicularis* (MBo, MAc, MI58, MI60, MI64, and MI66; 4.2–5.0 kg) were used for spontaneous behavioral evaluation in the lab-chair and/or home-cage contexts and two others (a female *Macaca mulatta*, MT 5.0 kg and a male *Macaca fascicularis*, MC4.5 kg) were used to assess the effect of VP injections on their performance during an approach-avoidance instrumental task (Figure 1A). The first six monkeys were also involved in a previous study on the effects of VP injections on positron emission tomography (PET) imaging (Galineau et al., 2017), while the other two performing the approach-avoidance task were involved in a VP neuronal recording study with the same task (Saga et al., 2016). Animal care and housing were compliant with National Institutes of Health guidelines (1996), the European Communities Council Directive of 2010 (2010/63/UE), and the French National Committee (87/848) recommendations.

During the behavioral experiments, the animals were seated in two types of primate chair. The first type is completely open, and the animal is free to move its limbs, allowing evaluation of spontaneous behavior (see Figure 3A). In the second, more closed type, only an opening at left- or right-hand level enabled access to a touch screen in front of the animal to perform the instrumental task. In both cases, a device implanted in the animal’s head allowed injection of the GABAergic agents directly into the VP by descending a cannula through the dura mater. After 3–12 months of training, depending on the protocol (spontaneous or task-motivated context), a plastic chamber and head holder were fixed to the monkey’s skull under general anesthesia and in sterile conditions. Positioning of the chamber allowing injections into the VP was estimated using structural MRI scans (1.5T; CEA-Orsay or CERMEP-Bron, France). The center of the MRI-compatible chamber was aligned based on the anterior commissure (AC), the best anatomical marker to allow penetration into the VP. The behavioral and injection systems have been more extensively described in our previous studies (Saga et al., 2016; Galineau et al., 2017). Finally, heart rate is one of the physiological markers of emotional state which could be reflect a negative emotional state such as worry or stress (Hofmann et al., 2005; Fisher and Newman, 2013). We hypothesized muscimol or bicuculline could have effects on behavioral state reflected in changing physiological state. To examine this, an implant (Data Science International, MN, USA) to monitor heart rate during the task and the VP.

**Figure 2:**
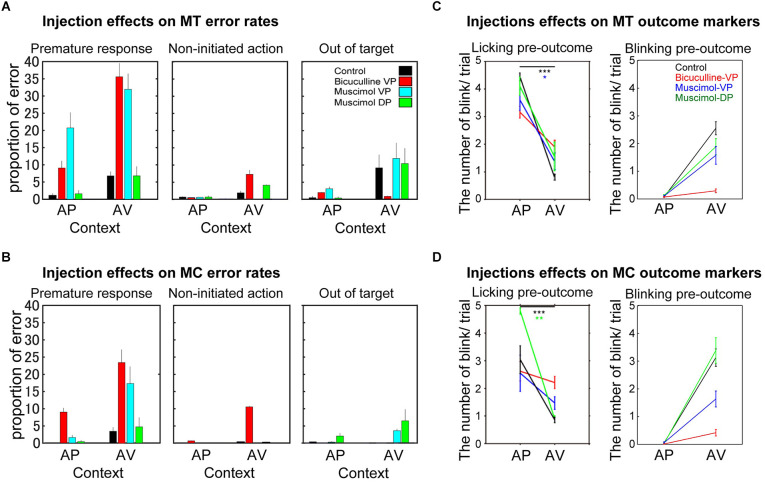
Different behavioral effects induced by bicuculline and muscimol injections into the Pallidum on three error types **(A,B)** and two expected outcome markers **(C,D)** observed during task performance for both monkeys (**A** and **C** for MT; **B** and **D** for MC) in appetitive (AP) and aversive (AV) contexts. **(A,B)** The panels indicate the average proportion (mean ± SEM) of premature response (left), non-initiated action (middle), and the out-of-target errors (right). The histogram bars show the control session (black), bicuculline into the VP (red), muscimol into the VP (cyan), and muscimol into the DP (green). **(C,D)** The panels indicate the number of licking movements (left) and the probability of eyeblinking during the pre-outcome period, when the monkeys anticipate the outcome of their choice in each context (AP or AV). As in parts **(A)** and **(B)**, the black, red, blue, and green marks indicate control injections, bicuculline into the VP, muscimol into the VP, and muscimol into the DP, respectively. The asterisks indicate statistical significances; *p < 0.05, **p < 0.01, ***p < 0.001.

**Figure 3:**
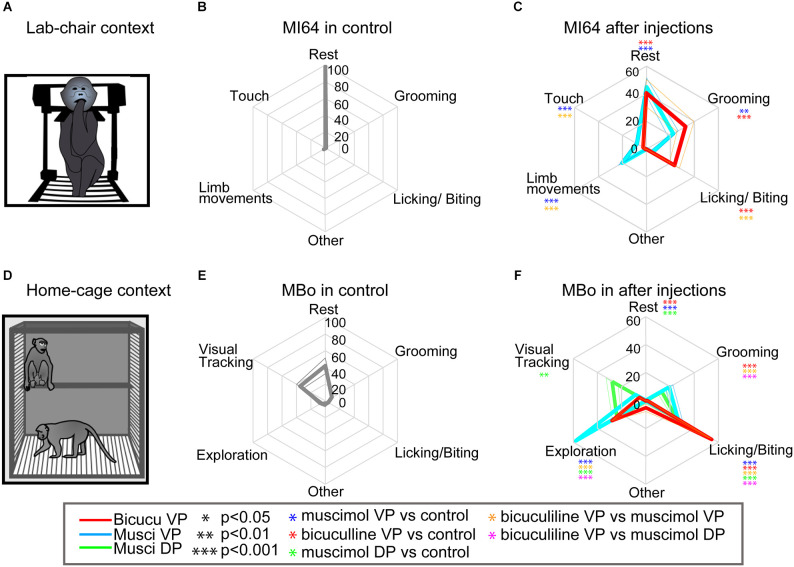
Experimental conditions and behavioral patterns during chair and home-cage evaluation. **(A)** Illustration of the lab-chair context with an animal expressing finger licking/biting. A monkey sitting down on the chair with its neck held loosely, allowing it to move its head and limbs (arms and legs) in the chair. **(B)** An example of monkey MI64’s behavioral patterns in the lab-chair context. The spider chart indicates average behavioral patterns during control sessions (13 sub-period). The axis indicates the average spent time (seconds) as a percentage among monkeys. Behavioral patterns in the lab-chair were categorized as rest, grooming, licking/biting, touch, limb movement, and other. **(C)** The spider chart indicating monkey MI64’s average behavioral patterns in the lab-chair context with injections (13 sub-periods for each injection). The axis indicates average spent time during behavioral session (mean ± SEM). The colored asterisks indicate statistical significances compared to the associated control session after injection; *p < 0.05, **p < 0.01, ***p < 0.001. **(D)** Illustration of the home-cage context with an animal expressing two different behaviors: visual tracking while sitting in the upper part of the cage and exploration behaviors by moving around in the lower part of the cage. In this context, the monkeys were totally free to move about without any restriction. **(E,F)** MBo’s behavioral patterns following injection in the home cage with **(E)**. An example of MBo’s behavioral patterns in the home-cage context (12 sub-period). The spider chart indicates average behavioral patterns during control sessions. Behavioral patterns in the lab-chair were categorized as rest, grooming, licking/biting, visual tracking, exploration, and other. **(F)** The spider chart indicating MBo’s average behavioral patterns in the home-cage context following injections (12 sub-periods for each injection). The colored asterisks have the same signification as in panel **(C)**.

### Bicuculline and muscimol microinjections

**Figure 4:**
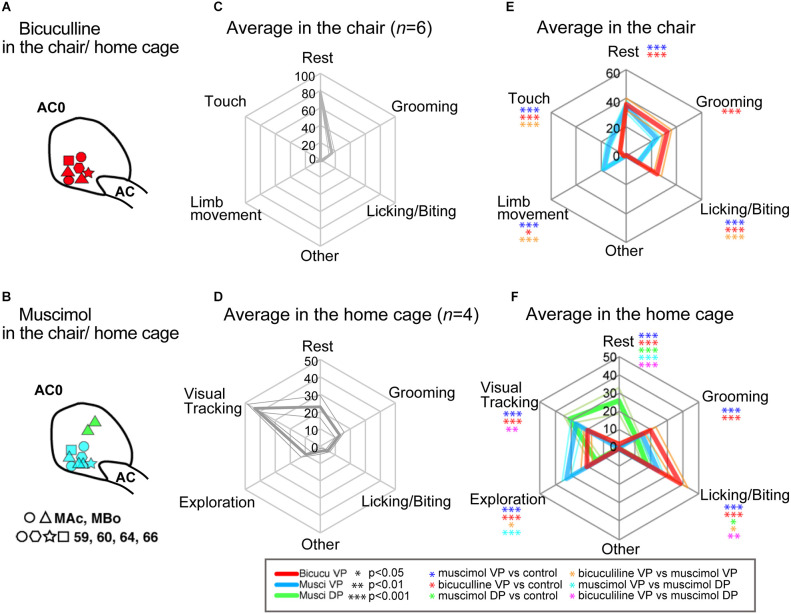
Injection sites for all monkeys and average behavioral patterns in the lab-chair and home-cage contexts. **(A)** Bicuculline injection sites at AC0 level. Each mark is associated with each monkey. **(B)** Similar representation for Muscimol into the VP and DP. **(C)** Average behavioral patterns (*n* = 6 monkeys) in the lab-chair context after control saline injections. The axis indicates the average spent time as a percentage among monkeys. **(D)** Average behavioral patterns (*n* = 4) in the home-cage context following control injections. **(E)** Average behavioral patterns in the lab-chair context following the different injections (bicuculline into the VP: *n* = 7 and muscimol into the VP: *n* = 8). Each color indicates behavioral changes following bicuculline and muscimol injections. Thick lines indicate average time spent of behavioral patterns and thin lines show the standard error of mean (SEM). **(F)** Average behavioral pattern following injection (*n* = 4 monkeys) in the home cage (bicuculline into the VP: *n* = 3 and muscimol into the VP: *n* = 4, and muscimol into the DP: *n* = 2). The numbers on the axis shown in the graph indicated the percentage of measured time that the monkeys spent performing the listed behaviors.

Before the microinjections, the location of VP was determined using MRI and neuronal activity recordings. This last approach enabled the depth separating the VP from the cortical surface to be estimated and the structures crossed by the electrode before reaching the VP to be identified. The anterior pallidum was specifically targeted from 1 mm anterior and posterior to the AC (for more details, see Saga et al., 2016, 2017). Bicuculline and muscimol were injected using a 30-gauge cannula tube connected to a 10 μl microsyringe (Hamilton). Bicuculline methiodide (volume 1.5–1.7 μl; concentration 15 μg/μl; Grabli et al., 2004), muscimol methiodide (volume 2.0–2.5 μl; concentration 1 μg/μl), or saline (volume 2.0 μl) were injected at 1.0 μl/min into the VP, with muscimol also being injected into the dorsal part of the anterior pallidum (DP; Figures 1B,C, 4A,B). After placing the cannula tube in the targeted position, the monkeys started with a pre-injection (P0) session (20 min with the cannula tube inserted but no injection). Then, experimental sessions started with a pharmacological injection and the behavioral task beginning 5 min after each injection to ensure their effects on neuronal activity. The subsequent behavioral sessions were defined corresponding to the time after injection; P1 5–25 min, P2: 25–45 min, and P3: 45–65 min after injection (Saga et al., 2016). The animals’ performances during injection were compared to a pre-injection session (P0) as well as to sessions on control days (without injection or with saline injection), with respect to each of the post-injection periods (P1 to P3) to determine the latency of appearance of a significant effect. The cannula tube remained in place throughout the whole experiment to minimize leaking outside the targeted structure and prevent backflow of the substance (from VP to DP). At the end of the session, each cannula guide was carefully removed, enabling each monkey to go back to its home cage. To observe a completely free-moving context in the home cage, the injection was performed at the end of the lab session just before the animal returned to its home cage. The monkeys were given an injection a maximum of two times per week. The remaining days were used as control days without injections or for neuronal recordings. Injection sites were defined as “highly effective” and “weakly effective” when behavioral effects appeared between 5 and 25 min and between 26 and 45 min after injection, respectively.

### Self-initiated behaviors in free-moving contexts

To assess behavioral changes in the chair and in the home cage, we used the same behavioral quantification and analysis procedure as in our previous studies (see Grabli et al., 2004; Worbe et al., 2009; Galineau et al., 2017). The behaviors frequently expressed in the chair context were resting (open eyes), grooming, finger licking/biting, touching the experimental equipment and limb movements (Figures 3B,C, 4C,E) while in the home-cage context, the most frequent were resting, visual tracking, grooming, finger licking/biting, and cage exploration (Figures 3E,F, 4D,F). For both contexts, we included less frequent behaviors in the other behaviors category. To avoid social interaction with other monkeys that could change the results, the monkeys was isolated in their home cage for 1 h post-injection. The duration of occurrences of each behavior was quantified after observation on recorded video in both contexts. In chair context, the behaviors were evaluated in 3 min segments over a 15 min control period (P0, *n* = 5 measures) before microinjection and during the three post-injection periods (P1: 5–20 min, P2: 25–40 min, and P3: 45–60 min). Each of these post-injection periods consisted of five behavioral measurements and were spaced by the performance of a simple food intake task of 5 min (results not shown). Since there was no food intake period after the return to the cage, we split the observation periods P1 to P3 into sub-periods of 5 min giving four measurements per period with the same post-injection time course as the other experimental contexts. P1: 5–25 min; P2: 25–45 min; and P3: 45–65 min. For all animals, control data in the home cage were obtained after control sessions in the chair. The effects of the injections were always compared to control days close to the injection and with respect to the periods P1 to P3 for the statistical comparisons.

The monkey was isolated in the home cage after injection to minimize social interaction with other monkeys. The duration of occurrences of each behavior was quantified by video recording in the chair and home cages with sub-period of 3 min each in the chair and 5 min in the cage from 5 min after injection. The experiments continued for 30 min in the chair and 60 min in the cage after injections. The sub periods could be variable depending on removal of the injection canula. The behavioral evaluations in the chair and home cage were performed on different days.

### Task-motivated behaviors in appetitive and aversive contexts

This study used the same delayed-response tasks used in our previous study (Saga et al., 2016), which comprised a single-cue condition (Figure 1A). A session of the single-cue task consisted of 70 trials (for Monkey C) or 100 trials (for Monkey T). In the single-cue task, 60% (i.e., 42 or 60) of the trials were appetitive (AP) and 40% (i.e., 28 or 40) were aversive (AV). To implement these proportions, no more than two appetitive trials were presented consecutively in the single-cue task. In sum, a bar was installed at waist level in front of the chair, and a 19-inch color video monitor equipped with a touch-sensitive screen was placed in front of the monkey (27 cm from its eyes). Eye movement and position were monitored at 120 Hz using an infrared eye-tracking system. Licking movement was monitored using infrared light. The presentation (Neurobehavioral systems, Inc, MA, USA) and scenario manager (Institut des Sciences Cognitives Marc Jeannerod, CNC, Bron, France) was used to control the behavioral task. The behavioral data were collected at 1,000 Hz using a Spike2 data acquisition system (Cambridge Electronic Design Ltd., CB, England). A liquid reward (apple juice: 0.2 ml/drop) was delivered *via* a small plastic hole placed in front of the monkey’s mouth. Single puffs of air delivered at 1.5–2.0 bar (25–35 psi) served as the primary aversive stimulus directed to the left side of the monkey’s face (cheek and eye) and delivered through a tube with its opening set at a distance of 10–15 cm from the face.

Three different types of subject error were possible, and trials were categorized accordingly. First, trials in which monkeys released the bar before the response target appeared on the screen were categorized as premature responses leading to interruption of the trial, with the conditioned stimulus (CS) disappearing immediately, followed by the inter-trial interval. Second, trials in which monkeys produced no response at all during the 2-s target presentation were categorized as non-initiated actions. Third, errors in which monkeys touched a region outside of the target area were categorized as errors related to a visuo-motor problem. Behavioral outcomes such as the number of complete trials, error trials, and the number of approach and avoidance behaviors were counted per session (i.e., P1–P3) during the task in each motivational context (AP and AV). The proportion of each outcome was then calculated. In addition to these behavioral parameters, the number of blinking 0.5 s before receiving the unconditioned stimulus (US) and the number of licking behavior (for 2.0 s) before receiving the US were measured in both monkeys (anticipation period, see detail in Saga et al., 2016 and Figures 2C,D). As well, heart rate was measured in MT (Supplementary Figure 1).

### Statistical analysis

A one-way analysis of variance (ANOVA; *p* = 0.05) was also performed to compare approach and avoidance behavior for each behavioral context between injection types. As for error type (i.e., the proportion of premature response, non-initiated action, and touch outside of the target in each behavioral session), a two-way ANOVA was performed with injection type (i.e., control, muscimol into the VP or DP, and bicuculline into the VP) and behavioral context (i.e., AP or AV) as factors. Then, a *post-hoc* Tukey test was performed to compare each group. Concerning the chair and home-cage evaluations, the time spent on each behavior was calculated and analyzed by converting each behavior duration to percentage based on sub-period (i.e., duration in seconds/180 s in the chair and behavior duration in seconds/300 s in the cage) using two tailed *t*-test (*p* < 0.05, Bonferroni-corrected). To investigate the difference regarding behavioral patterns associated with different injections, the cluster analysis was performed using the data obtained in the cage and chair and Ward’s clustering algorithm with Euclidean distance measure Metaboanalyst 5.0 (www.metaboanalyst.ca).

## Results

### Effects of bicuculline and muscimol microinjections on approach-avoidance behaviors

We first examined behavioral effects of bicuculline injections into the VP (Figure 1A) and muscimol injections into the VP and DP (Figures 1B,C) in the approach-avoidance instrumental tasks. In control sessions, they showed approach behavior in more than 95% of trials in the appetitive context (18 sessions in MT and 12 sessions in MC, Figure 1D, Table 1). On the other hand, the monkeys avoided the target associated with the aversive US in more than 60% of trials in the aversive context.

**Table 1:** The proportion of correct responses during behavioral task.

**Approach in AP**	**Control**	**Bicuculline VP**	**Muscimol VP**	**Muscimol DP**
Monkey T	96.1% ± 1.0%	84.7% ± 2.0%	83.6% ± 5.8%	95.8% ± 3.8%
Monkey C	95.2% ± 0.1%	89.8% ± 0.7%	98.6% ± 0.6%	97.9% ± 3.4%
Avoidance in AV	Control	Bicuculline VP	Muscimol VP	Muscimol DP
Monkey T	67.5% ± 2.6%	52.5% ± 3.3%	58.5% ± 4.2%	59.9% ± 2.7%
Monkey C	61.1% ± 1.6%	45.9% ± 1.5%	59% ± 1.7%	52.9% ± 3.1%

Mean ± SEM.

We performed 12 microinjections of bicuculline and eight injections of muscimol into the VP for two animals (MT and MC), and for comparison, eight injections of muscimol into the DP (Figures 1B,C). As for control, saline injections were performed three times into the VP and two times into the DP. These microinjections examined behavioral performances upon muscimol injection into the DP (*n* = 15 sessions in MT, *n* = 9 sessions in MC), muscimol injection into the VP (*n* = 14 sessions in MT, *n* = 9 sessions in MC), and bucuculline injection into the VP (*n* = 20 sessions in MT, *n* = 13 sessions in MC). Most of the injections induced strong effects especially in the VP, beginning at short latencies, 5–25 min after injection.

Both monkeys showed significant effects of injections on avoidance behaviors (*n* = 67 sessions, *F*_(3,63)_ = 2.96, *p* = 0.038 in MT and *F*_(3,39)_ = 9.24, *p* = 0.00009 in MC analyzed *n* = 43 sessions with a one-way ANOVA). Tukey’s HSD Test for multiple comparisons found a significant difference between control and bicuculline into the VP (*p* = 0.00009) and between muscimol into the VP and bicuculline into the VP (*p* = 0.0003) in MC, but not for MT. Although there were significant differences in approach behavior in the appetitive context in MC (*n* = 43 sessions, *F*_(3,39)_ = 2.98, *p* = 0.04), approach behaviors in appetitive context in MT remained the same following each injection (*n* = 67 sessions, *F*_(3,63)_ = 1.94, *p* = 0.13, Figure 1D).

MT showed significant error responses in the AV vs. AP context in control sessions (Figure 2A, Table 2, *F*_(1,35)_ = 8.9, *p* = 0.005), but this is not the case for MC (*F*_(1,23)_ = 0.003, *p* = 0.95).

**Table 2:** The proportion of error per session.

**Monkey T**		**Premature response**	**Non-initiated action**	**Out of tg**
Control	AP	1.2% ± 0.7%	0.4% ± 0.3%	0.3% ± 0.2%
	AV	5.8% ± 1.9%	4.7% ± 1.0%	9.1% ± 4.6%
Bicuculline VP	AP	9.1% ± 2.6%	0.6% ± 0.5%	2.1% ± 1.3%
	AV	35.6% ± 4.9%	7.8% ± 2.2%	0.9% ± 0.6%
Muscimol VP	AP	21% ± 4.4%	0.5% ± 0.3%	3% ± 1.3%
	AV	33.8% ± 5.5%	0.3% ± 0.2%	12.5% ± 3.7%
Muscimol DP	AP	1.7% ± 1.0%	0.7% ± 0.4%	0.4% ± 0.3%
	AV	6.8% ± 2.7%	4.4% ± 1.2%	10.3% ± 6.6%
Monkey C		Premature response	Non-initiated action	Out of tg
Control	AP	0.3% ± 0.3%	0.0%	1.1% ± 0.8%
	AV	3.6% ± 2.2%	0.2% ± 0.2%	0.1% ± 0.1%
Bicuculline VP	AP	9.0% ± 2.3%	0.7% ± 0.3%	0.0%
	AV	23.5% ± 4.0%	11.4 ± 2.5	0.0%
Muscimol VP	AP	1.6% ± 1.0%	0.0%	0.9% ± 0.9%
	AV	17.3% ± 5.3%	0.3% ± 0.2%	4% ± 1.6%
Muscimol DP	AP	0.4% ± 0.4%	0.0%	2.2% ± 0.9%
	AV	4.7% ± 2.9%	0.2% ± 0.2	7.0% ± 3.4%

Mean ± SEM.

We performed a two-way ANOVA for error responses with the injection type and context as factors. In both monkeys, erroneous trials increased significantly with injection type (*F*_(3,133)_ = 7.4, *p* < 0.0001, *F*_(3,85)_ = 9.7, *p* < 0.0001, for MT and MC, respectively), context factors (*F*_(1,133)_ = 32.2, *p* < 0.0001, *F*_(1,85)_ = 16.7, *p* < 0.0001 for MT and MC), and with interaction effect (*F*_(3,85)_ = 3.5, *p* < 0.03 in MC). *Post-hoc* test revealed a significant increase in errors following bicuculline injection into the VP compared to control (*p* < 0.005 and *p* < 0.0001, MT and MC) and following muscimol injection into the DP in both monkeys (*p* < 0.05 and *p* < 0.001).

For MT, *post-hoc* test showed a significant difference in muscimol into the VP as compared to control (*p* < 0.001) and muscimol into the DP (*p* < 0.03). Subsequently, we checked the proportion of each error response type in each session with two-way ANOVA (Figure 2, i.e., premature response, non-initiated action, and out of target). Significant effects in premature response were shown with both injection type (*F*_(3,133)_ = 16.4, *p* < 0.0001) in MT and *F*_(3,85)_ = 11.7, *p* < 0.0001 in MC) and context (*F*_(1,133)_ = 17.2, *p* < 0.0001) in MT and *F*_(1,85)_ = 11.5, *p* < 0.001 in MC). The interaction effect was confirmed only in MT (*F*_(3,133)_ = 4.0, *p* < 0.01). Increases in premature responses were significantly different following both bicuculline and muscimol injections into the VP compared to control and muscimol injection into the DP in both monkeys (Figures 2A,B, *post-hoc* Tukey test, *p* < 0.05). As for non-initiated actions, both monkeys showed significant effects of context (*F*_(1,133)_ = 17.2, *p* < 0.0001 in MT, *F*_(1,85)_ = 9.1, *p* < 0.003 in MC). MC showed interaction effect (*F*_(1,85)_ = 3.6, *p* < 0.03). Contrary to bicuculline, muscimol injected into the DP and VP tended to induce out-of-target errors (touches outside the target on-screen). Two-way ANOVA showed the effect of context (*F*_(1,133)_ = 5.0, *p* < 0.03 in MT, *F*_(1,85)_ = 4.9, *p* < 0.03 in MC) and interaction effect (*F*_(3,133)_ = 3.1, *p* < 0.03 in MT).

These results suggest that bicuculline and muscimol injections into the VP strongly influence behavioral patterns in the AV context but these were expressed differently.

Analysis of behavioral markers specific to the appetitive (licking movement before the juice drop) and aversive (eye blinking before the puff of air) contexts shows that both monkeys knew and anticipated the outcomes adapted to both contexts (Figures 2C,D, Table 3). Both monkeys showed context effect (*F*_(1,98)_ = 212.0, *p* < 0.0001 in MT, *F*_(1,78)_ = 114.0, *p* < 0.0001 in MC) and interaction effect (*F*_(3,98)_ = 11.0, *p* < 0.0001 in MT, *F*_(3,78)_ = 11.3, *p* < 0.0001 in MC) on licking during the pre-outcome period. *Post-hoc* test revealed significant difference between muscimol into the DP and control (*p* = 0.0365) and between bicuculline into the VP and control (*p* = 0.0076).

**Table 3:** The average proportion of number of licking/biting per trial.

	**Context**	**Control**	**Bicuculline VP**	**Muscimol VP**	**Muscimol DP**
**Licking**
Monkey T	AP	4.5 ± 0.1	3.2 ± 0.2	3.6 ± 0.4	4.1 ± 0.3
	AV	0.6 ± 0.1	1.7 ± 0.3	1.1 ± 0.2	1.0 ± 0.2
Monkey C	AP	3.1 ± 0.6	2.8 ± 0.3	4.8 ± 0.1	4.8 ± 0.1
	AV	0.6 ± 0.1	2.4 ± 0.3	1.2 ± 0.2	0.8 ± 0.1
**Blinking**					
Monkey T	AP	0.1 ± 0.1	0.1 ± 0.1	0.1 ± 0.1	0.1 ± 0.1
	AV	2.6 ± 0.2	0.3 ± 0.1	1.6 ± 0.3	1.9 ± 0.3
Monkey C	AP	0.1 ± 0.1	0.1 ± 0.1	0.1 ± 0.1	0.1 ± 0.1
	AV	3.1 ± 0.3	0.4 ± 0.1	1.6 ± 0.3	3.4 ± 0.5

The analysis of the number of blinks during pre-outcome period by two-way ANOVA showed significant effects on injection (*F*_(3,399)_ = 9.8, *p* < 0.0001 in MT, *F*_(3,399)_ = 11.2, *p* < 0.0001 in MC), context (*F*_(1,399)_ = 68.9, *p* < 0.0001 in MT, *F*_(1,399)_ = 82.4, *p* < 0.0001 in MC), and interaction *F*_(3,399)_ = 8.1, *p* < 0.0001 in MT, *F*_(3,399)_ = 11.5, *p* < 0.0001 in MC). The number of blinks in both monkeys showed a significant difference between muscimol into the DP and the VP, bicuculline into the VP and control (*p* < 0.0001), and between bicuculline into the VP and muscimol into the DP and VP (*p* < 0.0001).

We previously found that bicuculline injections into the VP induce significant increases in heart rate during task performance (Saga et al., 2016). In this study we found that only bicuculline injections into the VP induced gradual increases in heart rate (Supplementary Figure 1, *p* < 0.001) with moderate correlation of error responses (*r* = 0.33, *p* = 0.11). This result suggests that the effect of injecting bicuculline into the VP specifically influences the physiological state. Moreover, analysis of injection effects on outcome anticipation behavioral markers (Figures 2C,D) shows that bicuculline injections into the VP significantly altered both animals’ outcome anticipation of negative events in the AV context.

Together, bicuculline and muscimol injections into the VP greatly disturbed performance in approach-avoidance instrumental tasks, altering task-motivated behaviors. More specifically, injecting bicuculline into the VP produced more non-initiated actions or omissions to response, whereas muscimol injections induced more out-of-target errors, both in aversive contexts. Overall, injections into the DP induced fewer effects than other injections. These behavioral effects during the task suggest that different motivational contexts induce heterogeneous abnormal behaviors by acting in opposite ways on GABA transmission into the VP and the neighboring pallidal territory (DP).

### Effect of bicuculline and muscimol microinjections on spontaneous behaviors

To further clarify the abnormal behaviors observed in the approach-avoidance tasks, we investigated how bicuculline and muscimol injections modify spontaneous behaviors in the lab chair or in a totally free-moving context after the monkeys return to their home cage (Figures 3A,D). In total, 17 injections (Figures 4A,B, bicuculline into the VP: *n* = 7 and muscimol into the VP: *n* = 8, and muscimol into the DP: *n* = 2) were performed with six other monkeys (Monkey 59, 60, 64, 66, MBo, and MAc) in the chair and in home cage (MBo and MAc). Figure 3B shows the example of behavioral patterns in the chair without injection for monkey M64, which spent almost all its time staying and resting in the chair. The other monkeys also spent most of their time resting (Figure 4C). In the home cage, MBo spent most of the time gazing somewhere or resting (Figure 3E). Another monkey showed a similar behavioral profile, including grooming, exploration and finger licking/biting (Figure 4D).

Injecting either muscimol or bicuculline into the VP profoundly affected monkey behaviors. Individual examples of injection effects are given in Figures 3C,F while behavioral effects at the group scale are shown in Figures 4E,F. Microinjections decreased each monkey’s resting time and induced two behavioral profiles. The first profile is characterized by a strong statistical increase compared to control sessions in time spent in grooming and licking/biting finger behaviors (two tailed t-test, df = 5, *p* < 0.001). This profile was observed in both contexts (chair and home cage) and especially for licking/biting for bicuculline injections into the VP (see Figures 3E,F, 4E,F). The second profile featured a statistical increase compared to control sessions of several behaviors, with a strong significant (two tailed *t*-test, df = 5, *p* < 0.001) increase of limb movements and behaviors related to exploring in the cage including touching equipment in the chair, visual tracking and exploration by walking around in the cage (see Figures 3C–F, 4E,F). This hyperactivity profile was observed in both contexts (chair and home cage) and unlike the first profile induced by bicuculline injections into the VP, this second profile is mainly induced by muscimol injections into both the VP and DP. To provide objective evidence, a cluster analysis for spontaneous behavior was performed. The cluster analysis showed two different behavioral patterns between bicuculline- and muscimol-injected monkeys in the cage (Supplementary Figure 2A). This clustering was observed in behavioral patterns in the chair condition among six monkeys (Supplementary Figure 2B). The main difference between muscimol injections into the ventral (VP) or dorsal (DP) portions of the anterior pallidum is that injections into the VP induced exploration by walking around inside the homecage, while injections into the DP mainly induced visual exploration in the NHPs, i.e., looking around without moving their body.

Taken together, the effects observed in both the task-motived contexts and self-initiated behavior contexts showed that muscimol injections into the VP can induce a variety of behavioral markers related to impulsive disorders with hyperactivity. On the other hand, bicuculline injection into the VP was characterized by excessive reactions in the AV task context and compulsive behaviors such as grooming and self-biting observed in both free-moving contexts.

## Discussion

In this study, we found that motivational behaviors were disturbed by injecting both bicuculline and muscimol into the VP in non-human primates. Although these behavioral alterations with both GABAergic agents injected into the VP induced similar effects in task-motivated contexts, the effects of these two agents injected during the free-moving context induced different behavioral profiles. The GABAergic inactivation by bicuculline injections into the VP induced excessive reactions in the AV task context and anxiety-related behaviors such as grooming and self-biting, suggesting an anxious profile, while GABAergic activation with muscimol led to behavioral hyperactivity characteristic of an impulsive profile. Moreover, injecting muscimol into the DP was less effective in producing behavioral alteration than injection into the VP, which more strongly modified the appetitive and aversive task-motivated behaviors.

### Behavioral disorders can be induced by opposite GABAergic dysfunctions in the VP

It should be noted that the injection volumes we tested differed between muscimol (2.0–2.5 μl) and bicuculline (1.5 μl). We also tested injection of 1.5 μl of muscimol into the VP, but few effects were observed (data not shown). A previous study by Tachibana and Hikosaka (2012) performed muscimol injection with a reward-biased saccade task. They obtained effects with bilateral injections of 1.0–2.0 μl. However, injections in only one hemisphere showed weaker effects or none. In contrast, injection of 1.5 μl of bicuculline consistently and sufficiently exhibits behavioral effects (Grabli et al., 2004; Saga et al., 2016). Therefore, we speculated that blocking GABA_A_ signals may induce stronger effects than activating them. Previously, neuronal recordings confirmed the diffusion of bicuculline injections in the anterior striatum (Worbe et al., 2009). In this study, bicuculline’s effects on neuronal activity appeared in 10 min with a 1.5 μl injection at 0.6 mm from the injection site. In addition, neuronal recordings 1 mm from the injection site after injecting 3.0 μl of bicuculline showed effects on neuronal activity an average of only 23 min after injection. Most of the effects produced by the microinjections started 5 min post-injection (Figures 1B–E). Moreover, injecting muscimol into the DP compared to the VP led to distinct effects, suggesting that these observed behavioral effects were due to local changes in the neuronal activities of the pallidal territory where the injection was given.

### Opposite VP dysfunctions induced approach-avoidance disturbances in task-motivated contexts

Because muscimol and bicuculline are known to have opposite modes of action, leading to reduction and increase of VP neuronal activity, respectively, we expected that injecting each compound would produce opposite behavioral effects. However, our results indicated that both injections into the VP disturbed approach-avoidance behaviors, suggesting that GABAergic modulation in the VP plays crucial roles in approach-avoidance behavior or a range of aberrant neuronal activity leads to disturbances in approach-avoidance behavior. Both substances in the VP induced premature responses that were particularly accentuated in the AV context (Figures 2A,B). In addition, they showed different error types: bicuculline specifically induced non-initiated action in the AV context, while muscimol induced more out-of-target errors (Figures 2A,B). The effects in the AV context suggest that both activation and suppression of the VP activity control goal-directed behavior in negative motivational contexts. These results strongly support recent studies in rodents showing the involvement of VP GABA-neurons in motivation underlying risky choice (Farrell et al., 2021) by modulation of aversive processes (Wulff et al., 2019). Moreover, the effects of the injections on our outcome anticipation behavioral markers (licking movements and eye-blinking) show that bicuculline injections into the VP significantly disturbed the negative event outcome anticipation in both our animals in the AV context. Finally, only bicuculline injections into the VP increased the heart rate as already described (Supplementary Figure 1 and Saga et al., 2016), which is an internal physiological manifestation of a change in emotional or anxious state (Fisher and Newman, 2013). Although it is not clear how this specific alteration directly influences behavior, we previously showed that bicuculline injection into the VP influenced cortical activity in the anterior insula and amygdala (Galineau et al., 2017), two cerebral regions involved in negative value encoding (Zhang et al., 2013) and anxiety-related behaviors (Jensen et al., 2003; Delgado et al., 2009). Given that these activities change at the network level, the behavioral effects observed in this study could be due to abnormalities in the cortico-basal ganglia network, in particular in limbic-related areas (Saga et al., 2017). Altogether, these results suggest that both activation and suppression of the VP influence task-motivated behaviors by modulating a key node of a cortico-basal ganglia network more particularly involved in aversive contexts.

### Opposite VP dysfunctions in free-moving contexts induce impulsive-like and compulsive-like behaviors

To extend our findings, we also measured the spontaneous behaviors both injections elicited in the experimental chair without any task and in the monkey’s homecage (Figures 3, 4). In these contexts, muscimol and bicuculline injections both reduced resting time and led to distinct behavioral modifications, with the former inducing limb movements related to increased exploration and hyper locomotion, and the latter leading to increased finger licking/biting and grooming (Grabli et al., 2004). The intensity of the elicited behaviors also differed. Combining these results with the increased premature responses and out-of-target screen touches observed during the task strongly supports a hyperactive state induced by muscimol injection into the VP. Premature responses and out-of-target errors tended to increase in the AV context, suggesting disinhibition of erratic reactions in performing negative motivated behaviors (i.e., active avoidance to avoid the puff of air) following muscimol injections into the VP. On the other hand, the repetitive finger licking/biting or grooming elicited by bicuculline, along with the increased premature responses and non-initiated actions observed in the AV context during the task, suggest increased aversive reactions and a loss of aversive motivation related to activation of the VP. Repetitive actions or stereotypes could be expressed in inescapable situations or poor environments (Bryant et al., 1988; Novak et al., 1998). Moreover, heart rate acceleration following bicuculline injection could reflect modifications in the internal state that translate into an alteration of the physiological state. These behavioral effects, with or without motivational contexts and changes in physiological state, imply that activation of the VP may result in abnormal aversive information processing associated with excessive aversive behavior. Importantly, muscimol injection into the DP showed weaker effects (Figures 1C and 4F), and bicuculline injections into the associative part of the GPe (corresponding with the DP in this study) have previously been associated with attention problems and hyperactivity (Grabli et al., 2004). Due to the small size of the VP, its functional abnormalities are difficult to detect in clinical imaging studies. However, our study suggests that both excessive and reduced VP activation can lead to pathological states. For example, when looking at a picture associated with aversive events, patients with anxiety or phobia exhibited abnormal activation in the anterior insula and ventral striatum (Remijnse et al., 2006; Simmons et al., 2006, 2011), which connect directly and indirectly to the VP (Spooren et al., 1996; Chikama et al., 1997; François et al., 2004; Sgambato-Faure et al., 2016). Therefore, regulation of these pathways could modulate both appetitive and aversive motivational behaviors.

In conclusion, despite the evolution in size and functional organization of the VP between rodents and primates, our results in non-human primate confirm those obtained in rodents which shown the important role of the VP in the motivational processes underlying reward seeking as well as avoidance in aversive contexts (Creed et al., 2016; Heinsbroek et al., 2020; Moaddab et al., 2021; Liu et al., 2022). Our results obtained on non-human primate in different experimental contexts also show that opposite dysfunctions of VP activity could be involved in both human impulsive and compulsive disorders, which makes it a potential therapeutic target by acting on its GABAergic transmission.

## Data Availability Statement

The raw data supporting the conclusions of this article will be made available by the authors, without undue reservation.

## Ethics Statement

Animal care and housing were in compliance with the NIH guidelines (1996) and with the European Communities Council Directive of 2010 (2010/63/UE) recommendations. Procedures were approved by the French National Committee (#991-2015063017055778).

## Author Contributions

YS, LG, and LT: designed research, performed research, and wrote the manuscript. YS and LG: analyzed data. All authors contributed to the article and approved the submitted version.

## Supplementary Material

Supplementary Figure 1 Behavioral and physiological markers recorded after injections into the pallidum. Effect on heart rate change in monkey MT while performing behavioral tasks. The vertical axis indicates heart rate (beats per minute: bpm) and the horizontal axis indicates each session. Session 1, 2, and 3 indicate the time following injection, i.e., a 6–25 min, 26–45 min, and 46–65 min, respectively. The black, cyan, green, and red lines show changes in heart rate in the control, muscimol (into the VP and DP) and bicuculline (VP) injections.

Supplementary Figure 2 Dendrogram representation of cluster analysis after injections. **(A)** Hierarchical clustering of the behavioral responses observed for two monkeys in the home cage condition after either bicuculline or muscimol injections into the ventral or dorsal pallidum (VP and DP, respectively) displayed as a dendrogram representing Euclidean distances between clusters based on the Ward clustering algorithm (Metaboanalyst 5.0). **(B)** Hierarchical clustering of the behavioral responses observed for six monkeys in the chair condition after either bicuculline or muscimol injections in the ventral pallidum displayed as a dendrogram representing Euclidean distances between clusters based on the Ward clustering algorithm (Metaboanalyst 5.0).
