# Supplementary figures and images for: Impulsive and compulsive behaviors can be induced by opposite GABAergic dysfunctions inside the primate ventral pallidum
Source: Front Syst Neurosci. 2022 Dec 8;16:1009626. doi: 10.3389/fnsys.2022.1009626 (PMC9774472; doi:10.3389/fnsys.2022.1009626)

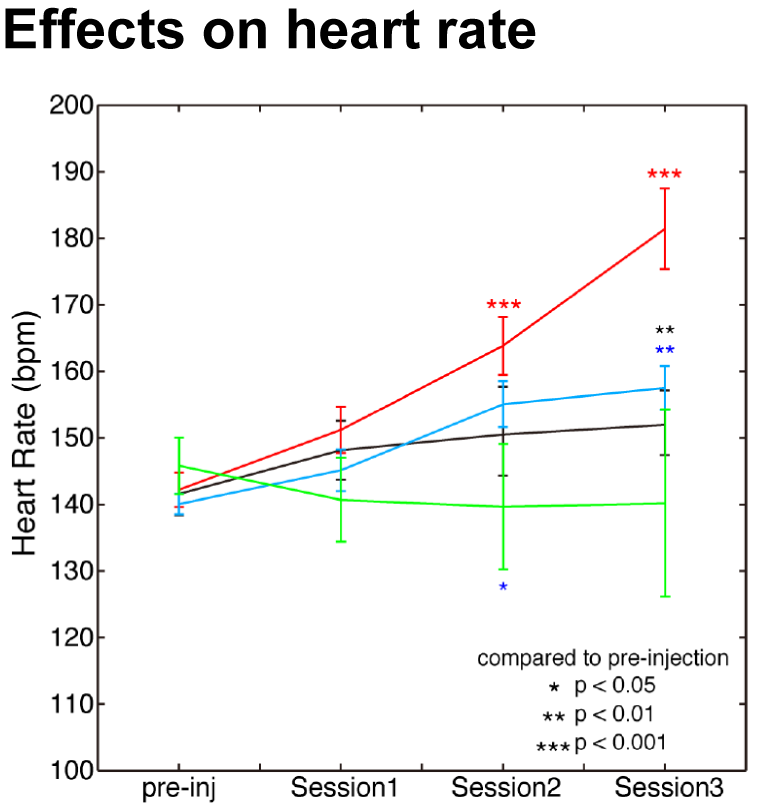

Supplement: Supplementary Figure 1 — Behavioral and physiological markers recorded after injections into the pallidum. Effect on heart rate change in monkey MT while performing behavioral tasks. The vertical axis indicates heart rate (beats per minute: bpm) and the horizontal axis indicates each session. Session 1, 2, and 3 indicate the time following injection, i.e., a 6–25 min, 26–45 min, and 46–65 min, respectively. The black, cyan, green, and red lines show changes in heart rate in the control, muscimol (into the VP and DP) and bicuculline (VP) injections. [file Image_1.tif]

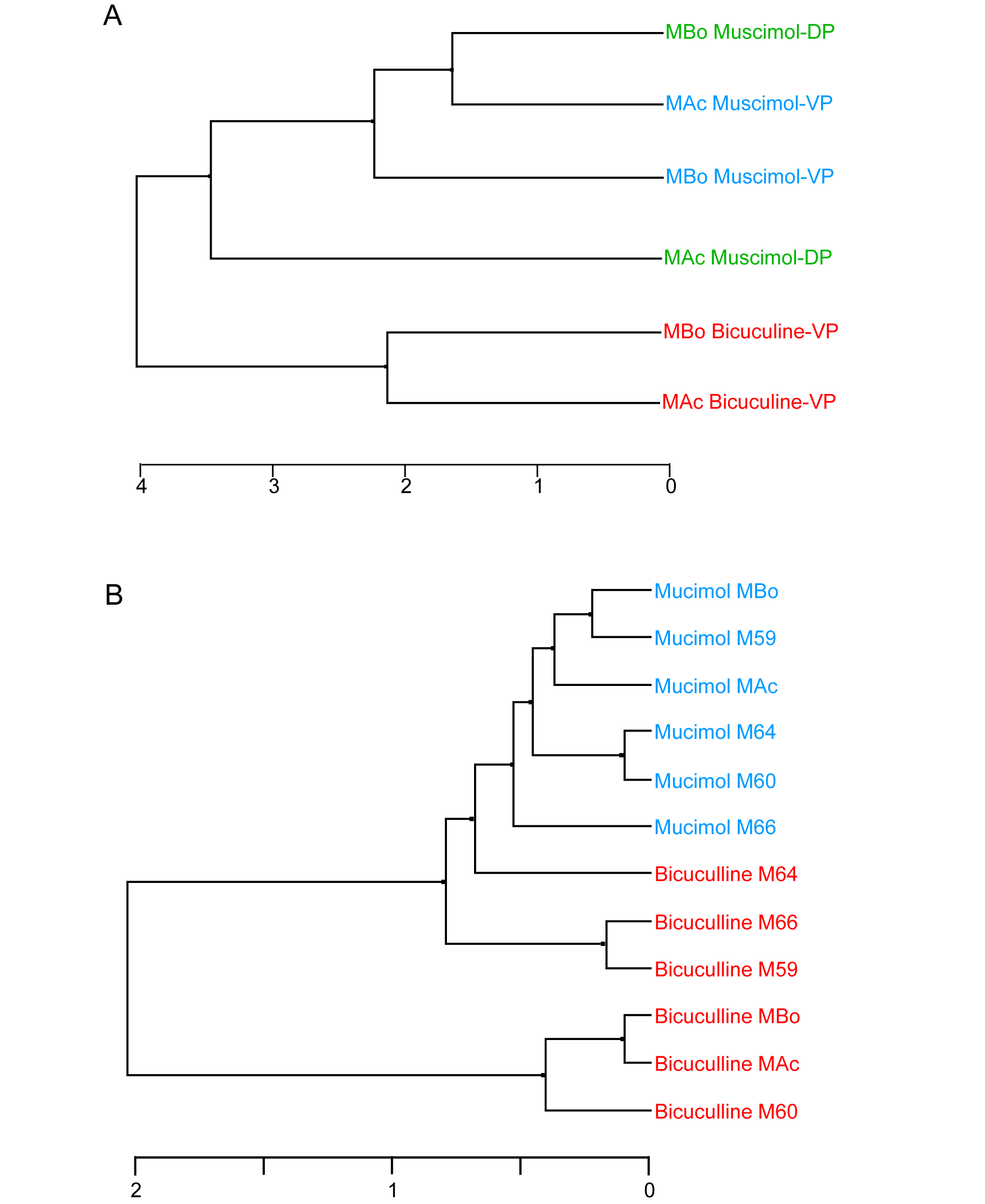

Supplement: Supplementary Figure 2 — Dendrogram representation of cluster analysis after injections. (A) Hierarchical clustering of the behavioral responses observed for two monkeys in the home cage condition after either bicuculline or muscimol injections into the ventral or dorsal pallidum (VP and DP, respectively) displayed as a dendrogram representing Euclidean distances between clusters based on the Ward clustering algorithm (Metaboanalyst 5.0). (B) Hierarchical clustering of the behavioral responses observed for six monkeys in the chair condition after either bicuculline or muscimol injections in the ventral pallidum displayed as a dendrogram representing Euclidean distances between clusters based on the Ward clustering algorithm (Metaboanalyst 5.0). [file Image_2.tif]
